# Influence of Insulin Resistance and TNF-*α* on the Inflammatory Process, Oxidative Stress, and Disease Activity in Patients with Rheumatoid Arthritis

**DOI:** 10.1155/2016/8962763

**Published:** 2016-05-31

**Authors:** Neide Tomimura Costa, Tatiana Mayumi Veiga Iriyoda, Ana Paula Kallaur, Francieli Delongui, Daniela Frizon Alfieri, Marcell Alysson Batisti Lozovoy, Ricardo Braga Amin, Vinicius Daher Alvares Delfino, Isaias Dichi, Andréa Name Colado Simão

**Affiliations:** ^1^Department of Rheumatology, University of Londrina (UEL), Londrina, PR, Brazil; ^2^Department of Clinical Analysis, University North of Paraná (UNOPAR), Londrina, PR, Brazil; ^3^Department of Clinical Pathology, University of Londrina, Robert Koch Avenue No. 60 Bairro Cervejaria, Londrina 86038-440, PR, Brazil; ^4^Department of Internal Medicine, University of Londrina, Londrina, PR, Brazil

## Abstract

The aim of this study was to evaluate the involvement of TNF-*α* and insulin resistance (IR) in the inflammatory process, oxidative stress, and disease activity in patients with rheumatoid arthritis (RA). This cross-sectional study included 270 subjects (control group, *n* = 97) and RA patients (*n* = 173). RA patients were divided into four groups: the first group without IR and not using antitumor necrosis factor-*α* (TNF−) (G1, IR− TNF−); the second group without IR and using anti-TNF-*α* (G2, IR− TNF+); the third group with IR and not using anti-TNF-*α* (G3, IR+ TNF−); and the fourth group with IR and using anti-TNF-*α* (G4, IR+ TNF+). G3 and G4 had higher (*p* < 0.05) advanced oxidation protein products (AOPPs) and oxidative stress index (OSI) compared to G1. G4 group presented higher (*p* < 0.05) AOPPs and OSI than G2. TRAP was significantly lower in G3 compared to G1. Plasma TNF-*α* levels were significantly higher in G4 and G2 compared to G1 (*p* < 0.0001) and G3 (*p* < 0.0001 and *p* < 0.01, resp.). The presence of insulin resistance was robustly associated with both oxidative stress and TNF-*α* levels. More studies are warranted to verify if IR can be involved in therapeutic failure with TNF-*α* inhibitors. This trial is registered with Brazilian Clinical Trials Registry Register number RBR-2jvj92.

## 1. Introduction

Rheumatoid arthritis (RA) is a chronic inflammatory disease that leads to severe joint destruction. In addition, RA patients have higher risk of developing cardiovascular disease (CVD) and this is related to chronic inflammation [1] and corticosteroids treatment [2, 3]. Systemic chronic inflammation and proinflammatory cytokines have been proposed as major protagonists in the pathogenesis of insulin resistance (IR), an important factor for CVD [4, 5]. TNF-*α* plays a central role in the pathogenesis of RA [6, 7] and has also been implicated in the development of IR [4, 8]. In addition, single infusion of the anti-TNF-*α* monoclonal antibody decreased insulin resistance in RA patients [9]. Abnormalities in glucose metabolism have been well documented in RA patients and may also correlate with Disease Activity Score evaluating 28 joints (DAS 28) [9].

Oxidative stress has a prominent role in the etiology and pathogenesis of joint tissue injury and chronic inflammation in patients with RA, which may lead to connective tissue degradation and joint and periarticular deformities [10]. Reactive oxygen species (ROS) have been considered an enhancer factor for autoimmune disease risk [11]. ROS are important intracellular signaling molecules in the cells of the immune system that amplify the synovial inflammatory-proliferative response [12]. Previous studies showed that elevated levels of lipoperoxidation and decreased antioxidant system in RA are positively correlated with DAS 28 and high sensitivity C-reactive protein (hsCRP) [13, 14]. Tumor necrosis factor-alpha (TNF-*α*) can induce higher oxidative stress by initiators of the nuclear factor kappa B activation cascade and is under its transcriptional control, constituting a positive feedback loop [11]. Moreover, anti-TNF-*α* therapy can reduce oxidative stress in patients with RA [15, 16].

Our group has investigated the development of IR and the metabolic syndrome in chronic inflammatory diseases [17–20] and these reports have found an important role of oxidative stress in the development and maintenance of these conditions. Therefore, it seems that chronic inflammation and oxidative stress contribute to the pathogenesis of both RA and IR. Furthermore, previous studies have shown that IR [8, 21–23] and oxidative stress [15, 16, 24–26], independently, may impair disease activity in patients with RA.

Therefore, the aim of the present study was to verify the influence of insulin resistance and TNF-*α* on the inflammatory process, oxidative stress, and disease activity in patients with RA.

## 2. Patients and Methods

### 2.1. Subjects

This cross-sectional study included 270 subjects, healthy individuals (control group, *n* = 97) and RA patients (*n* = 173), aged between 18 and 70 years. The control group was selected from among blood donors of the University Hospital who did not present autoimmune disease, and RA patients were selected from among the Ambulatory of Rheumatology of the University Hospital of Londrina, Paraná, Brazil. RA patients were initially divided into two groups: the first group without IR (IR−, *n* = 91) and the second group with IR (IR+, *n* = 82). After that, to verify the influence of insulin resistance and also of anti-TNF-*α* therapy on anthropometric, biochemical, immunological, and oxidative stress parameters in patients with RA, they were divided into four groups: the first group (control group) without IR and not using anti-TNF-*α* therapy (G1, IR−  TNF−, *n* = 71); the second group without IR and using anti-TNF-*α* therapy (G2, IR− TNF+, *n* = 20); the third group with IR and not using anti-TNF-*α* therapy (G3, IR+ TNF−, *n* = 63); and the fourth group with IR and using anti-TNF-*α* therapy (G4, IR+ TNF+, *n* = 19). RA patients (G2 and G4) were using anti-TNF-*α* therapy at least for six months. Sex, age, and ethnicity were controlled. RA was classified according to the 2010 rheumatoid arthritis classification criteria [27].

Disease activity status was determined using DAS 28 [9] and patients were classified into four different groups, namely, (1) remission group: DAS 28 ≤ 2.6; (2) low disease activity group: 2.6 < DAS 28 ≤ 3.2; (3) moderate disease activity group 3.2 < DAS 28 ≤ 5.1; and (4) high disease activity group: DAS 28 > 5.1.

None of the subjects was receiving a specific diet. The individuals of both groups (control and RA) did not smoke and did not drink alcohol regularly. None of the participants in the study presented heart, thyroid, renal, hepatic, gastrointestinal, or oncological diseases, and none were receiving estrogen replacement therapy or drugs for hyperlipidemia or hyperglycemia or antioxidant supplements. This study was conducted according to the guidelines laid down in the Declaration of Helsinki and the Ethical Committee of the University of Londrina, Paraná, Brazil, approved all procedures involving human subjects and patients. Written informed consent was obtained from all subjects/patients.

### 2.2. Anthropometric Measurements

Body weight was measured in the morning to the nearest 0.1 kg by using an electronic scale with individuals wearing light clothing and without shoes; height was measured to the nearest 0.1 cm by using a stadiometer. Body mass index was calculated as weight (kg) divided by height (m) squared. Waist circumference (WC) was measured on standing subjects midway between the lowest rib and the iliac crest.

### 2.3. Biochemical, Immunological, and Hematological Biomarkers

After fasting for 12 hours, serum or plasma samples were obtained and the patients underwent the following laboratory blood analysis: glucose and uric acid (UA) were evaluated by a biochemical autoanalyzer (Dimension Dade AR, Dade Behring®, Deerfield, IL, USA), using Dade Behring kits; plasma insulin level and anticyclic citrullinated peptide (anti-CCP) antibody were determined by chemiluminescence microparticle immunoassay (Architect, Abbott Laboratory, Abbott Park, IL, USA). The homeostasis model assessment-IR (HOMA-IR) was used as a surrogate measurement of insulin resistance [28]. Consider the following: HOMA-IR = insulin fasting (*μ*U/mL) × glucose fasting (nmol/L)/22.5. IR was considered when HOMA-IR ≥ 2.114 [8]. Serum high sensitivity CRP (hsCRP) and rheumatoid factor (RF) were measured using a nephelometric assay (Behring Nephelometer II, Dade Behring, Marburg, Germany). TNF-*α* levels were measured by a sandwich enzyme-linked immunosorbent assay (ELISA) using a commercial immunoassay ELISA (Ready-SET-Go! Set, e-Bioscience, San Diego, California, USA). Erythrocyte sedimentation rate (ESR) was obtained by automated kinetic-photometric method (Ves-Matic CUBE 30, DIESSE, Siena, Italy).

### 2.4. Oxidative Stress Measurements

Samples for evaluating oxidative stress and total antioxidant capacity were performed with EDTA as anticoagulant and antioxidant. All samples were centrifuged at 3.000 rpm for 15 minutes and plasma aliquots stored at −70°C until assayed.

### 2.5. Tert-Butyl Hydroperoxide-Initiated Chemiluminescence (CL-LOOH)

The CL-LOOH in plasma was evaluated as described previously by Gonzalez Flecha et al. [29]. For chemiluminescence (CL) measurement, reaction mixtures were placed in 20 mL scintillation vials (low-potassium glass) containing final concentrations of plasma (250 *μ*L), 30 mM KH_2_PO_4_/K_2_HPO_4_ buffer (pH 7.4), and 120 mM KCl with 3 mM of tert-butyl hydroperoxide in a final volume of 2 mL. Tert-butyl hydroperoxide-initiated chemiluminescence was measured in Beckman LS 6000 Liquid Scintillation Counter set to the out-of-coincidence mode, with a response range from 300 to 620 nm. The vials were kept in the dark up to the moment of assay, and determination was carried out in a dark room at 30°C. The results are expressed in counts per minute (cpm).

### 2.6. Determination of Advanced Oxidation Protein Products (AOPPs)

AOPPs were determined in the plasma using the semiautomated method described by Witko-Sarsat et al. [30]. AOPPs results of oxidation of amino acid residues such as tyrosine, leading to the formation of dityrosine-containing protein cross-linking products detected by spectrophotometry [17, 30]. AOPPs concentrations were expressed as micromoles per liter (*μ*mol/L) of chloramines-T equivalents.

### 2.7. Total Radical-Trapping Antioxidant Parameter (TRAP)

TRAP was determined as reported by Repetto et al. [31]. This method detects hydrosoluble and/or liposoluble plasma antioxidants by measuring the chemiluminescence inhibition time induced by 2,2-azobis(2-amidinopropane). The system was calibrated with vitamin E analog Trolox, and the values of TRAP are expressed in equivalent of *μ*M Trolox/mg UA. TRAP analysis in conditions associated with hyperuricemia, as in patients with MetS, may be jeopardized because uric acid concentration is responsible for 60% of plasma total antioxidant capacity. Thus, a correction of total antioxidant capacity based on uric acid concentration is needed [32, 33].

### 2.8. Oxidative Stress Index (OSI)

Oxidative stress imbalance was verified when OSI was calculated as AOPPs (*μ*mol/L) divided by TRAP (*μ*M Trolox/mg UA), which indicates the oxidant-antioxidant ratio as a reflection of the cellular redox state.

### 2.9. Statistical Analysis

Distribution of sex, ethnicity, and therapy was analyzed by chi-square test with Yates correction. Comparisons between groups were performed using the Kruskal-Wallis test with Dunn's posttest and data were expressed as the median (25–75%). The results were considered significant when *p* < 0.05. To determine which factors were independently associated with IR in RA patients, the variables that presented *p* < 0.10 in univariate analyses were included in logistic regression model. Logistic regression analyses were performed with SPSS v20.0 (IBM, USA).

## 3. Results

Rheumatoid arthritis patients with or without IR were not statistically different in relation to disease duration and serum RF and anti-CCP levels and frequency in prednisone and antimalarials and methotrexate and leflunomide use and anti-TNF-*α* therapy (Table 1). However, IR+ group had an increased DAS 28 (*p* = 0.043) with enhanced frequency in patients with high disease activity. In addition, IR+ group showed higher ESR (*p* = 0.023) and hsCRP (*p* = 0.040) compared to the IR− group (Table 1).

With regard to anthropometric and biochemical markers, IR+ group presented higher BMI (*p* < 0.0001, *p* < 0.0001), WC (*p* < 0.01; *p* < 0.0001), plasma glucose (*p* < 0.0001, *p* < 0.0001), and insulin (*p* < 0.0001, *p* < 0.0001) levels and HOMA-IR (*p* < 0.0001, *p* < 0.0001) compared to the control group and IR− group, respectively (Table 2).

**Figure 1:**
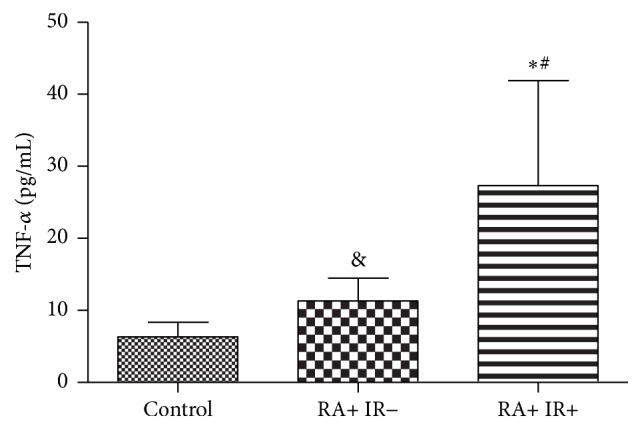
Plasma TNF-*α* levels in healthy subjects (controls) and in patients with rheumatoid arthritis with (IR+) or without (IR−) insulin resistance. Kruskal-Wallis test with Dunn's posttest. ^*∗*^IR+ versus control, *p* < 0.0001; ^&^IR− versus control, *p* < 0.01; ^#^IR+ versus IR−, *p* < 0.05.

In relation to oxidative stress markers, both IR− and RI+ groups had significantly higher OSI (*p* < 0.0001) compared to the control group, whereas IR− group showed lower AOPPs (*p* < 0.05) levels compared to the control group. Higher AOPPs (*p* < 0.0001) and OSI (*p* < 0.001) and lower TRAP (*p* < 0.05) were verified in the group composed of IR+ patients in relation to IR− group (Table 2). Plasma TNF-*α* levels were significantly higher both in IR− (*p* < 0.01) and in IR+ (*p* < 0.0001) groups compared to the control group (Figure 1). In addition, RI+ group had higher plasma TNF-*α* levels than IR− group (*p* < 0.05) (Figure 1).

**Figure 2:**
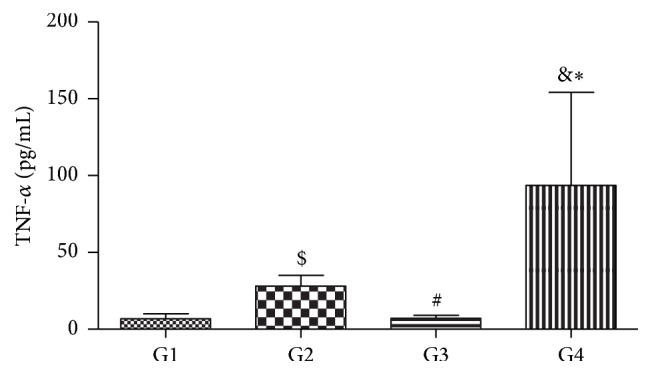
Plasma TNF-*α* levels in patients with rheumatoid arthritis with (IR+) or without (RI−) insulin resistance and using (TNF+) or not using (TNF−) anti-TNF-*α*. Kruskal-Wallis test with Dunn's posttest. G1: IR− TNF−; G2: IR− TNF+; G3: IR+ TNF−; and G4: IR+ TNF+. ^*∗*^G4 versus G1, *p* < 0.0001; ^&^G4 versus G3, *p* < 0.0001; ^$^G2 versus G1, *p* < 0.0001; and ^#^G2 versus G3, *p* < 0.01.

Table 3 shows the differences when the groups were divided taking into account the presence or absence of IR and anti-TNF-*α* therapy. The groups composed of patients with IR, IR+ TNF− (G3) and IR+ TNF+ (G4), had higher (*p* < 0.05) AOPPs and OSI compared to G1 (control group). In addition, G4 group presented higher (*p* < 0.05) AOPPs and OSI than IR− TNF+ (G2) group. TRAP was significantly lower in IR+ TNF− group (G3) in relation to G1. On the other hand, the groups without insulin resistance, G1 and G2, showed no differences in oxidative stress markers (Table 3). In relation to the inflammatory profile, ESR showed significantly higher (*p* < 0.05) levels in G3 and G4 than in G1, and G4 had also increased ESR (*p* < 0.05) levels compared to G2. There were significantly lower (*p* < 0.05) hsCRP levels in G4 compared to G3 (Table 3). Plasma TNF-*α* levels were significantly higher in patients who were using anti-TNF-*α* therapy, that is, G4 (*p* < 0.0001) and G2 (*p* < 0.0001), compared to G1 (Figure 2). Also, G4 and G2 had higher plasma TNF-*α* levels than G3 (*p* < 0.0001 and *p* < 0.01, resp.) (Figure 2).

Oxidative stress data according to anti-TNF-*α* therapy with etanercept or adalimumab are shown in Table 4. There was no significant difference in CL-LOOH, AOPPs, TRAP, or OSI values. However, AOPP levels showed an increase trend (*p* = 0.071) in patients using adalimumab and this trend was independent of BMI (*p* = 0.047, OR: 1.009, CI 95%: 1.000–1.018) (data not shown). In sum, presence of IR was related to increase in DAS 28 and ESR and hsCRP and TNF-*α* levels and AOPPs and OSI and decreased TRAP in patients with RA. On the other hand, IR did not have a role in changes related to RF and anti-CCP. In addition, TNF-*α* increase is related to IR development in patients with RA.

## 4. Discussion

Several reports have shown that IR is related to chronic inflammation [1, 32, 33] and corticosteroid treatment [2, 3]. Although previous articles have shown that corticosteroid may be involved in IR [21, 22, 34], this finding has not been verified in patients with RA, suggesting that corticosteroid beneficial anti-inflammatory effects would compensate the deleterious metabolic action [35, 36]. Penesová et al. [36] showed that low-dose glucocorticoid treatment with duration of 2–9 years is relatively safe and did not lead to glucose metabolism impairment. Independently of whether they had IR or not, in the present study the patients did not differ in the frequency they were using prednisone, showing that, in this cohort of RA patients, corticosteroid use does not seem to be a determinant factor for IR development. Moreover, patients used less than 7.5 mg/d corticosteroid (data not shown), which has been reported as safe [37].

Several reports have shown the association between chronic inflammatory disease states and IR [32, 33, 38]. Previous studies demonstrated that TNF-*α* may have an important role in the IR pathogenesis by multiple mechanisms, such as downregulation of genes that are required for normal insulin action, direct effects on insulin signaling, induction of elevated free fatty acids via stimulation of lipolysis, and negative regulation of peroxisome proliferator-activated receptor-*γ* (PPAR*γ*), an important insulin-sensitizing nuclear receptor [39]. In RA patients with severe and active disease even in the presence of anti-TNF-*α* therapy, high-grade inflammation was correlated negatively and independently with circulating adiponectin concentration [40], an important anti-inflammatory adipokine related to insulin resistance and metabolic syndrome [41].* In vitro* studies have shown that TNF-*α* induced serine phosphorylation of insulin receptor substrate-1 (IRS-1) and inhibited insulin receptor tyrosine kinase, causing a change of the insulin signaling [40]. In the present study, patients using anti-TNF-*α* therapy, which is generally indicated to patients who have a severe disease not controlled by disease-modifying antirheumatic drugs (DMARDs), showed higher TNF-*α* levels. Even with anti-TNF-*α* therapy, TNF-*α* levels have not reached the values obtained by patients who control disease activity with conventional therapy and DAS 28 maintained higher score (≥3.3) than the recommended one for patients using or not using biological agents [41]. Of note, the majority of our patients (66.7%), who were taking anti-TNF-*α* therapy, used etanercept, a soluble TNF-*α* receptor fusion protein. Etanercept prolongs the half-life of TNF-*α* with a subsequent rise in measured serum TNF-*α* levels; thus it renders TNF-*α* biologically inactive and unavailable to bind to its receptor [42–44]. In the current study, patients with IR had also higher ESR concomitantly to TNF-*α* increase, suggesting that chronic inflammatory process may be associated with IR development and maintenance in patients with RA. Regarding the present data, it is not possible to assure that etanercept changed TNF-*α* in a biologically inactive substance. However, it is conceivable to suggest that other proinflammatory cytokines, which were not evaluated in this study, may be involved in the inflammatory process verified in patients with IR.

The present study demonstrated that RA patients with IR have higher TNF-*α* levels and unfavorable oxidative status. Reactive oxygen species (ROS) damage directly cellular elements in cartilage and either directly or indirectly the components of the extracellular matrix by upregulating mediators of matrix degradation. ROS impair chondrocyte response to growth factors and migration to sites of cartilage injury. In addition, ROS inhibit the synthesis of matrix components including proteoglycans by chondrocytes [12]. In the present study, IR patients showed higher oxidative stress levels and DAS 28. The overproduction of TNF-*α* is thought to be the main contributor to increased ROS release in RA patients [24, 45, 46], leading to tissue damage and IR [47, 48]. Large amounts of ROS have been detected in the synovial fluid in RA [49], and this production can be induced by TNF-*α* stimulation [50]. TNF-*α* exerts its cytotoxic effects via generation of intracellular ROS that induce apoptosis [51, 52]. Moreover, TNF-*α* can induce ROS production from neutrophils through pathway activating phagocytic NADPH oxidases in mitochondria [53] and TNF-*α* combined with cytokines such as GM-CSF or G-CSF enhances O_2_^−^ generation [54]. Of note, oxidative stress and IR are more closely associated and many evidences have shown that oxidative stress can lead to IR by promoting the expression of several proinflammatory cytokines, mainly TNF-*α*, interleukin 6 (IL-6), and interleukin 17 (IL-17), which can cause significant decline in insulin sensitivity [9]. On the other hand, ROS may increase TNF-*α* levels because they function as a second messenger to stimulate nuclear factor kappa B dependent expression of proinflammatory cytokines [55]. Altogether, our data seem to suggest that higher TNF-*α* level can be involved in IR development and maintenance and have a direct influence on oxidative stress. It seems that a cyclic and complex relationship occurs between TNF-*α*, oxidative stress, and IR in patients with RA.

The administration of biological drugs seems to have a role in increasing the barrier which the body possesses against oxidative stress [56]. However, data about anti-TNF-*α* therapy remain a matter of controversy. Kageyama et al. [15] showed a decrease in oxidative stress markers after six months in 22 patients with RA using etanercept. In contrast, den Broeder et al. [46] did not find any significant changes in oxidative stress markers after two weeks in 21 patients with RA taking adalimumab, although marked reduction in neutrophil influx to synovial tissue with anti-TNF-*α* therapy was reported. Meanwhile, Biniecka et al. [45] evaluated oxidative stress, assessed by 4-hydroxy-2-nonenal (4-HNE) in the synovial tissue, after three months in 18 patients with RA using anti-TNF-*α* therapy. DAS 28 < 2.6 was found in seven patients who were considered as anti-TNF-*α* responders and DAS 28 ≥ 2.6 in 11 patients who were considered as anti-TNF-*α* nonresponders. There was a decrease in 4-HNE levels only in anti-TNF-*α* responders patients. The aforementioned study seems to suggest that anti-TNF-*α* therapy can decrease oxidative stress in RA patients by controlling the inflammatory process, and hence they do not act directly on the production of ROS. In the present study, most patients who used anti-TNF-*α* therapy were taking etanercept. Nevertheless, differently from Kageyama's et al. study [15], the patients did not show improvement in redox state. It is conceivable to suggest that this may have occurred because anti-TNF-*α* therapy maintained DAS 28 in similar values obtained by patients who were not using anti-TNF-*α* therapy. Meanwhile, inflammatory process shown by increased ESR and TNF-*α* levels, mainly in RA patients with IR, progressed in these patients being responsible for oxidative stress increase.

Upon looking at the results obtained in the present study, some limitations have to be considered. First, the cross-sectional design does not allow for inference causality. Second, although the minimum number of patients has been reached by the calculation of the sample size, a greater number of patients would probably confer more strength to the statistical results.

This study corroborates with Binieckas et al.'s [45], which suggested that inflammatory state maintenance can be responsible for oxidative stress found in patients with RA. On the other hand, the data of the present study show that IR is involved in an unbalanced redox state, which possibly contributes to maintaining a vicious circle of high-grade inflammation.

## 5. Conclusions

This study demonstrates that IR and TNF-*α* are important factors involved in redox imbalance in patients with RA and it seems to be due to the maintenance of inflammatory state and disease activity. The data from the present study suggest a complex interaction of TNF-*α*, oxidative stress, and IR, but the presence of insulin resistance seems to be directly associated with both oxidative stress and TNF-*α* levels. The differences in oxidative stress markers in RA patients with or without IR could contribute to a better design for future drugs and/or nutritional interventional studies in this population. In addition, more studies are warranted to verify if IR can be involved in therapeutic failure with TNF-*α* inhibitors.

## Figures and Tables

**Table 1:** Clinical and laboratory data in patients with rheumatoid arthritis with (IR+) or without (IR−) insulin resistance.

	IR− (*n* = 91)	IR+ (*n* = 82)	*p*
Disease duration (years)	11.0 (5.0–18.3)	8.0 (4.0–20.3)	NS
RF (IU/mL)	48.3 (0.0–125.0)	26.9 (0.0–118.2)	NS
Anti-CCP (U/mL)	25.55 (0.13–120.10)	6.65 (0.50–131.40)	NS
DAS 28	3.51 (2.39–4.49)	3.76 (2.85–4.78)	**0.043**
DAS 28, *n* (%)			
Remission (<2.6)	27 (29.7% )	16 (19.5%)	
Low (2.6–3.2)	12 (13.2%)	11 (13.4%)	**0.001**
Moderate (3.2–5.1)	42 (46.1%)	39 (47.6%)	
High (>5.1)	10 (10.0%)	16 (19.5%)	
CPR (mg/L)	3.52 (1.31–12.38)	6.35 (2.51–11.08)	**0.040**
ESR (mm)	14.0 (6.0–22.0)	19.5 (9.3–35.5)	**0.023**
*Therapy*			
Prednisone (Y/N)	64/27	54/28	NS
Antimalarials (Y/N)	38/53	32/50	NS
Anti-TNF-*α* (Y/N)	20/71	19/63	NS
Adalimumab	7	6	NS
Etanercept	13	13	
Methotrexate (Y/N)	57/34	62/20	NS
Leflunomide (Y/N)	40/51	35/47	NS

Chi-square test with Yates correction. Mann-Whitney test. Data are expressed as median (25–75%). Y, yes; N, no; RF, rheumatoid factor; anti-CCP, anti-cyclic citrullinated peptide antibody; DAS 28, Disease Activity Score evaluating 28 joints; CRP, C-reactive protein; ESR, erythrocyte sedimentation rate; and NS, not significant.

**Table 2:** Anthropometric, clinical, and laboratorial profile in healthy subjects (controls) and in patients with rheumatoid arthritis (RA) with or without insulin resistance (IR).

	Controls (*n* = 97)	RA+ IR− (*n* = 91)	RA+ IR+ (*n* = 82)	Control versus RA+ IR−	Control versus RA+ IR+	RA+ IR− versus RA+ IR+
Gender (F/M)	80/17	70/21	70/12	NS	NS	NS
Caucasian/not Caucasian	72/25	58/33	53/29	NS	NS	NS
Age (years)	51.0 (42.5–69.5)	56.0 (46.0–63.3)	57.5 (48.8–62.3)	NS	NS	NS
BMI (kg/m^2^)	25.8 (23.8–28.0)	25.9 (22.8–29.3)	29.4 (25.3–33.4)	NS	**<0.0001**	**<0.0001**
WC (cm)	91.5 (87.0–97.3)	90.0 (82.0–97.3)	98.0 (91.0–107.3)	NS	**<0.01**	**<0.0001**
Glucose (mg/dL)	87.0 (82.8–95.0)	85.0 (80.0–90.0)	96.0 (88.9–113.0)	NS	**<0.0001**	**<0.0001**
Insulin (*μ*U/mL)	6.35 (4.60–8.03)	6.70 (5.30–8.10)	13.95 (11.10–16.78)	NS	**<0.0001**	**<0.0001**
HOMA-IR	1.35 (1.01–1.69)	1.42 (1.07–1.75)	3.41 (2.71–4.46)	NS	**<0.0001**	**<0.0001**
CL-LOOH (cpm)	166.7 (141.9–179.0)	169.2 (150.0–198.9)	166.2 (152.6–201.5)	NS	**NS**	**NS**
AOPP (*μ*mol/L of chloramines-T equivalents)	150.4 (118.4–209.6)	123.5 (100.4–171.3)	173.8 (123.9–238.7)	<0.05	**NS**	**<0.0001**
TRAP (*μ*M Trolox/mg UA)	158.9 (122.2–200.9)	171.5 (146.1–207.9)	155.9 (121.0–177.3)	NS	**NS**	**<0.05**
OSI	0.228 (0.166–0.321)	0.762 (0.578–0.952)	1.183 (0.753–1.680)	<0.0001	**<0.0001**	**<0.001**

Kruskal-Wallis test with Dunn's posttest. Data are expressed as median (25–75%). BMI, body mass index; WC, waist circumference; HOMA-IR, homeostasis model assessment-insulin resistance; CL-LOOH, tert-butyl hydroperoxide-initiated chemiluminescence; AOPPs, advanced oxidation protein products; TRAP, total radical-trapping antioxidant parameter; and OSI, oxidative stress index.

NS: not significant.

**Table 3:** Oxidative stress markers, disease activity, and inflammatory parameters in patients with rheumatoid arthritis with (IR+) or without (IR−) insulin resistance and using (TNF+) or not using (TNF−) anti-TNF-*α*.

	G1 (*n* = 71)	G2 (*n* = 20)	G3 (*n* = 63)	G4 (*n* = 19)
CL-LOOH (cpm)	170.7 (150.0–196.7)	167.4 (147.2–214.4)	165.7 (152.7–204.3)	166.2 (151.8–166.2)
AOPP (*μ*mol/L of chloramines-T equivalents)	124.5 (102.6–170.1)	123.2 (99.9–182.8)	173.3^*∗*^ **(122.4–242.7)**	173.8^#&^ **(124.4–222.4)**
TRAP (*μ*M Trolox/mg UA)	175.4 (147.3–210.0)	164.7 (131.8–207.7)	150.8^*∗*^ **(121.0–178.8)**	159.2 (107.5–176.6)
OSI	0.73 (0.57–0.92)	0.85 (0.62–1.12)	1.21^*∗*^ **(0.78–1.79)**	1.18^#&^ **(0.69–1.53)**
DAS 28	3.41 (2.23–4.57)	3.83 (3.08–4.89)	3.75 (2.87–4.80)	3.49 (2.78–4.30)
CRP (mg/dL)	4.74 (1.26–15.80)	2.75 (1.78–6.76)	6.63 (7.70–11.9)	4.66^*∗∗*^ **(1.42–8.89)**
ESR (mm)	14.0 (5.0–22.0)	14.5 (8.3–23.0)	19.0^*∗*^ **(8.0–32.5)**	26.0^&#^ **(11.0–44.0)**

Kruskal-Wallis test with Dunn's posttest. Data are expressed as median (25–75%). G1, IR− TNF−; G2, IR− TNF+; G3, IR+ TNF−; G4, IR+ TNF+; CL-LOOH, tert-butyl hydroperoxide-initiated chemiluminescence; AOPP, advanced oxidation protein product; TRAP, total radical-trapping antioxidant parameter; OSI, oxidative stress index; DAS 28, Disease Activity Score evaluating 28 joints; CRP, C-reactive protein; and ESR, erythrocyte sedimentation rate.

^*∗*^G1 versus G3, *p* < 0.05; ^#^G1 versus G4, *p* < 0.05; ^&^G4 versus G2, *p* < 0.05; and ^*∗∗*^G4 versus G3.

**Table 4:** Oxidative stress in patients with rheumatoid arthritis using adalimumab or etanercept.

Parameters	Etanercept *n* = 26	Adalimumab *n* = 13	*p*
CL-LOOH (cpm)	164.02 (145.51–187.05)	168.06 (162.71–197.90)	NS
AOPP (*μ*mol/L of chloramines-T equivalents)	127.44 (108.75–187.05)	167.80 (122.90–228.73)	0.071
TRAP (*μ*M Trolox/mg UA)	157.27 (15.84–183.51)	159.70 (148.83–175.58)	NS
OSI	1.10 (0.77–1.31)	0.86 (0.74–1.37)	NS

Mann-Whitney test. Data are expressed as median (25–75%). CL-LOOH, tert-butyl hydroperoxide-initiated chemiluminescence; AOPP, advanced oxidation protein product; TRAP, total radical-trapping antioxidant parameter; OSI, oxidative stress index; and NS, not significant.

## Abbreviations

Anti-CCP: Anticyclic citrullinated peptide
AOPPs: Advanced oxidation protein products
CL-LOOH: Tert-butyl hydroperoxide-initiated chemiluminescence
hsCRP: Highly sensitive C-reactive protein
DAS 28: Disease Activity Score evaluating 28 joints
4-HNE: 4-Hidroxy-2-nonenal
CVD: Cardiovascular disease
DMARDs: Disease-modifying antirheumatic drugs
ESR: Erythrocyte sedimentation rate
HOMA-IR: Homeostasis model assessment-insulin resistance
IL-6: Interleukin 6
IL-17: Interleukin 17
IR: Insulin resistance
OSI: Oxidative stress index
PPAR*γ*: Peroxisome proliferator-activated receptor-*γ*
RA: Rheumatoid arthritis
RLU: Relative luminescence units
RF: Rheumatoid factor
ROS: Reactive oxygen species
TRAP: Total radical-trapping antioxidant parameter
TNF-*α*: Tumor necrosis factor-alpha
UA: Uric acid
WC: Waist circumference.

## References

[1] del Rincón I. D., Williams K., Stern M. P., Freeman G. L., Escalante A. (2001). High incidence of cardiovascular events in a rheumatoid arthritis cohort not explained by traditional cardiac risk factors. *Arthritis and Rheumatism*.

[2] Gabriel S. E. (2008). Cardiovascular morbidity and mortality in rheumatoid arthritis. *The American Journal of Medicine*.

[3] Haque S., Mirjafari H., Bruce I. N. (2008). Atherosclerosis in rheumatoid arthritis and systemic lupus erythematosus. *Current Opinion in Lipidology*.

[4] De Luca C., Olefsky J. M. (2008). Inflammation and insulin resistance. *FEBS Letters*.

[5] El-Barbary A. M., Kassem E. M., El-Sergany M. A. S., Essa S. A.-M., Eltomey M. A. (2011). Association of anti-modified citrullinated vimentin with subclinical atherosclerosis in early rheumatoid arthritis compared with anti-cyclic citrullinated peptide. *The Journal of Rheumatology*.

[6] Saxne T., Palladino M. A., Heinegard D., Talal N., Wollheim F. A. (1988). Detection of tumor necrosis factor *α* but not tumor necrosis factor *β* in rheumatoid arthritis synovial fluid and serum. *Arthritis and Rheumatism*.

[7] McInnes I. B., Schett G. (2011). The pathogenesis of rheumatoid arthritis. *The New England Journal of Medicine*.

[8] Chung C. P., Oeser A., Solus J. F. (2008). Inflammation-associated insulin resistance: differential effects in rheumatoid arthritis and systemic lupus erythematosus define potential mechanisms. *Arthritis and Rheumatism*.

[9] Prevoo M. L. L., van't Hof M. A., Kuper H. H., van Leeuwen M. A., van de Putte L. B. A., van Riel P. L. C. M. (1995). Modified disease activity scores that include twenty-eight-joint counts development and validation in a prospective longitudinal study of patients with rheumatoid arthritis. *Arthritis & Rheumatism*.

[10] Vasanthi P., Nalini G., Rajasekhar G. (2009). Status of oxidative stress in rheumatoid arthritis. *International Journal of Rheumatic Diseases*.

[11] Filippin L. I., Vercelino R., Marroni N. P., Xavier R. M. (2008). Redox signalling and the inflammatory response in rheumatoid arthritis. *Clinical and Experimental Immunology*.

[12] Hitchon C. A., El-Gabalawy H. S. (2004). Oxidation in rheumatoid arthritis. *Arthritis Research and Therapy*.

[13] Taysi S., Polat F., Gul M., Sari R., Bakan E. (2002). Lipid peroxidation, some extracellular antioxidants, and antioxidant enzymes in serum of patients with rheumatoid arthritis. *Rheumatology International*.

[14] Hassan S. Z., Gheita T. A., Kenawy S. A., Fahim A. T., El-Sorougy I. M., Abdou M. S. (2011). Oxidative stress in systemic lupus erythematosus and rheumatoid arthritis patients: relationship to disease manifestations and activity. *International Journal of Rheumatic Diseases*.

[15] Kageyama Y., Takahashi M., Nagafusa T., Torikai E., Nagano A. (2008). Etanercept reduces the oxidative stress marker levels in patients with rheumatoid arthritis. *Rheumatology International*.

[16] Shahmohamadnejad S., Vaisi-Raygani A., Shakiba Y. (2015). Association between butyrylcholinesterase activity and phenotypes, paraoxonase192 rs662 gene polymorphism and their enzymatic activity with severity of rheumatoid arthritis: correlation with systemic inflammatory markers and oxidative stress, preliminary report. *Clinical Biochemistry*.

[17] Lozovoy M. A. B., Simão A. N. C., Hohmann M. S. N. (2011). Inflammatory biomarkers and oxidative stress measurements in patients with systemic lupus erythematosus with or without metabolic syndrome. *Lupus*.

[18] Lozovoy M. A. B., Simão A. N. C., Oliveira S. R. (2013). Relationship between iron metabolism, oxidative stress, and insulin resistance in patients with systemic lupus erythematosus. *Scandinavian Journal of Rheumatology*.

[19] Oliveira S. R., Colado Simão A. N., Kallaur A. P. (2014). Disability in patients with multiple sclerosis: influence of insulin resistance, adiposity, and oxidative stress. *Nutrition*.

[20] Morimoto H. K., Simão A. N. C., Almeida E. R. D. D. (2014). Role of metabolic syndrome and antiretroviral therapy in adiponectin levels and oxidative stress in HIV-1 infected patients. *Nutrition*.

[21] Dessein P. H., Joffe B. I. (2006). Insulin resistance and impaired beta cell function in rheumatoid arthritis. *Arthritis and Rheumatism*.

[22] La Montagna G., Cacciapuoti F., Buono R. (2007). Insulin resistance is an independent risk factor for atherosclerosis in rheumatoid arthritis. *Diabetes and Vascular Disease Research*.

[23] Arcaro G. (2002). Insulin causes endothelial dysfunction in humans: sites and mechanisms. *Circulation*.

[24] Kageyama Y., Takahashi M., Ichikawa T., Torikai E., Nagano A. (2008). Reduction of oxidative stress marker levels by anti-TNF-*α* antibody, infliximab, in patients with rheumatoid arthritis. *Clinical and Experimental Rheumatology*.

[25] Altindag O., Karakoc M., Kocyigit A., Celik H., Soran N. (2007). Increased DNA damage and oxidative stress in patients with rheumatoid arthritis. *Clinical Biochemistry*.

[26] Nakajima A., Aoki Y., Shibata Y. (2014). Identification of clinical parameters associated with serum oxidative stress in patients with rheumatoid arthritis. *Modern Rheumatology*.

[27] Aletaha D., Neogi T., Silman A. J. (2010). 2010 rheumatoid arthritis classification criteria: An American College of Rheumatology/European League against rheumatism collaborative initiative. *Annals of the Rheumatic Diseases*.

[28] Haffner S. M., Miettinen H., Stern M. P. (1997). The homeostasis model in the San Antonio Heart Study. *Diabetes Care*.

[29] Gonzalez Flecha B., Llesuy S., Boveris A. (1991). Hydroperoxide-initiated chemiluminescence: an assay for oxidative stress in biopsies of heart, liver, and muscle. *Free Radical Biology and Medicine*.

[30] Witko-Sarsat V., Friedlander M., Khoa T. N. (1998). Advanced oxidation protein products as novel mediators of inflammation and monocyte activation in chronic renal failure. *The Journal of Immunology*.

[31] Repetto M., Reides C., Gomez Carretero M. L., Costa M., Griemberg G., Llesuy S. (1996). Oxidative stress in blood of HIV infected patients. *Clinica Chimica Acta*.

[32] Hotamisligil G. S. (2000). Molecular mechanisms of insulin resistance and the role of the adipocyte. *International Journal of Obesity*.

[33] Popa C., Netea M. G., Van Riel P. L. C. M., Van Der Meer J. W. M., Stalenhoef A. F. H. (2007). The role of TNF-*α* in chronic inflammatory conditions, intermediary metabolism, and cardiovascular risk. *Journal of Lipid Research*.

[34] Dessein P. H., Joffe B. I., Stanwix A. E., Christian B. F., Veller M. (2004). Glucocorticoids and insulin sensitivity in rheumatoid arthritis. *The Journal of Rheumatology*.

[35] Svenson K. L. G., Lundqvist G., Wide L., Hällgren R. (1987). Impaired glucose handling in active rheumatoid arthritis: effects of corticosteroids and antirheumatic treatment. *Metabolism*.

[36] Penesová A., Rádiková Z., Vlček M. (2013). Chronic inflammation and low-dose glucocorticoid effects on glucose metabolism in premenopausal females with rheumatoid arthritis free of conventional metabolic risk factors. *Physiological Research*.

[37] Sabio J. M., Vargas-Hitos J. A., Navarrete N., Hidalgo-Tenorio C., Jiménez-Alonso J. (2010). Effects of low or medium-dose of prednisone on insulin resistance in patients with systemic lupus erythematosus. *Clinical and Experimental Rheumatology*.

[38] Tam L.-S., Tomlinson B., Chu T. T., Li T. K., Li E. K. (2007). Impact of TNF inhibition on insulin resistance and lipids levels in patients with rheumatoid arthritis. *Clinical Rheumatology*.

[39] Moller D. E. (2000). Potential role of TNF-*α* in the pathogenesis of insulin resistance and type 2 diabetes. *Trends in Endocrinology and Metabolism*.

[40] Hayakawa T., Nagai Y., Taniguchi M. (2000). Tumor necrosis factor-*β* gene NcoI polymorphism decreases insulin resistance in Japanese men. *Metabolism: Clinical and Experimental*.

[41] Singh J. A., Furst D. E., Bharat A. (2012). 2012 update of the 2008 American College of Rheumatology recommendations for the use of disease-modifying antirheumatic drugs and biologic agents in the treatment of rheumatoid arthritis. *Arthritis Care & Research*.

[42] Lo J., Bernstein L. E., Canavan B. (2007). Effects of TNF-*α* neutralization on adipocytokines and skeletal muscle adiposity in the metabolic syndrome. *American Journal of Physiology—Endocrinology and Metabolism*.

[43] Grattendick K. J., Nakashima J. M., Feng L., Giri S. N., Margolin S. B. (2008). Effects of three anti-TNF-*α* drugs: etanercept, infliximab and pirfenidone on release of TNF-*α* in medium and TNF-*α* associated with the cell *in vitro*. *International Immunopharmacology*.

[44] Bhatia A., Kast R. E. (2007). Tumor Necrosis Factor (TNF) can paradoxically increase on etanercept treatment, occasionally contributing to TNF-mediated disease. *Journal of Rheumatology*.

[45] Biniecka M., Kennedy A., Ng C. T. (2011). Successful tumour necrosis factor (TNF) blocking therapy suppresses oxidative stress and hypoxia-induced mitochondrial mutagenesis in inflammatory arthritis. *Arthritis Research and Therapy*.

[46] den Broeder A. A., Wanten G. J. A., Oyen W. J. G., Naber T., Van Riel P. L. C. M., Barrera P. (2003). Neutrophil migration and production of reactive oxygen species during treatment with a fully human anti-tumor necrosis factor-*α* monoclonal antibody in patients with rheumatoid arthritis. *Journal of Rheumatology*.

[47] Stagakis I., Bertsias G., Karvounaris S. (2012). Anti-tumor necrosis factor therapy improves insulin resistance, beta cell function and insulin signaling in active rheumatoid arthritis patients with high insulin resistance. *Arthritis Research and Therapy*.

[48] Stavropoulos-Kalinoglou A., Metsios G. S., Panoulas V. F., Nightingale P., Koutedakis Y., Kitas G. D. (2012). Anti-tumour necrosis factor alpha therapy improves insulin sensitivity in normal-weight but not in obese patients with rheumatoid arthritis. *Arthritis Research and Therapy*.

[49] Biemond P., Swaak A. J. G., Koster J. F. (1984). Protective factors against oxygen free radicals and hydrogen peroxide in rheumatoid arthritis synovial fluid. *Arthritis and Rheumatism*.

[50] Woo C.-H., Kim T.-H., Choi J.-A. (2006). Inhibition of receptor internalization attenuates the TNF*α*-induced ROS generation in non-phagocytic cells. *Biochemical and Biophysical Research Communications*.

[51] Schulze-Osthoff K., Krammer P. H., Dröge W. (1994). Divergent signalling via APO-1/Fas and the TNF receptor, two homologous molecules involved in physiological cell death. *The EMBO Journal*.

[52] Hirose K., Longo D. L., Oppenheim J. J., Matsushima K. (1993). Overexpression of mitochondrial manganese superoxide dismutase promotes the survival of tumor cells exposed to interleukin-1, tumor necrosis factor, selected anticancer drugs, and ionizing radiation. *FASEB Journal*.

[53] Miesel R., Murphy M. P., Kröger H. (1996). Enhanced mitochondrial radical production in patients with rheumatoid arthritis correlates with elevated levels of tumor necrosis factor alpha in plasma. *Free Radical Research*.

[54] Mur E., Zabernigg A., Hilbe W., Eisterer W., Halder W., Thaler J. (1997). Oxidative burst of neutrophils in patients with rheumatoid arthritis: influence of various cytokines and medication. *Clinical and Experimental Rheumatology*.

[55] Bonizzi G., Piette J., Merville M.-P., Bours V. (2000). Cell type-specific role for reactive oxygen species in nuclear factor-kappaB activation by interleukin-1. *Biochemical Pharmacology*.

[56] Coaccioli S., Panaccione A., Biondi R. (2009). Evaluation of oxidative stress in rheumatoid and psoriatic arthritis and psoriasis. *Clinica Terapeutica*.

